# CD4 T Cell Responses and the Sepsis-Induced Immunoparalysis State

**DOI:** 10.3389/fimmu.2020.01364

**Published:** 2020-07-07

**Authors:** Matthew D. Martin, Vladimir P. Badovinac, Thomas S. Griffith

**Affiliations:** ^1^Department of Urology, University of Minnesota, Minneapolis, MN, United States; ^2^Department of Pathology, University of Iowa, Iowa City, IA, United States; ^3^Interdisciplinary Graduate Program in Immunology, University of Iowa, Iowa City, IA, United States; ^4^Department of Microbiology and Immunology, University of Iowa, Iowa City, IA, United States; ^5^Microbiology, Immunology, and Cancer Biology PhD Program, University of Minnesota, Minneapolis, MN, United States; ^6^Center for Immunology, University of Minnesota, Minneapolis, MN, United States; ^7^Masonic Cancer Center, University of Minnesota, Minneapolis, MN, United States; ^8^Minneapolis VA Healthcare System, Minneapolis, MN, United States

**Keywords:** CD4 T cell, sepsis, immunoparalysis, adaptive immunity, therapy

## Abstract

Sepsis remains a major cause of death in the United States and worldwide, and costs associated with treating septic patients place a large burden on the healthcare industry. Patients who survive the acute phase of sepsis display long-term impairments in immune function due to reductions in numbers and function of many immune cell populations. This state of chronic immunoparalysis renders sepsis survivors increasingly susceptible to infection with newly or previously encountered infections. CD4 T cells play important roles in the development of cellular and humoral immune responses following infection. Understanding how sepsis impacts the CD4 T cell compartment is critical for informing efforts to develop treatments intended to restore immune system homeostasis following sepsis. This review will focus on the current understanding of how sepsis impacts the CD4 T cell responses, including numerical representation, repertoire diversity, phenotype and effector functionality, subset representation (e.g., Th1 and Treg frequency), and therapeutic efforts to restore CD4 T cell numbers and function following sepsis. Additionally, we will discuss recent efforts to model the acute sepsis phase and resulting immune dysfunction using mice that have previously encountered infection, which more accurately reflects the immune system of humans with a history of repeated infection throughout life. A thorough understanding of how sepsis impacts CD4 T cells based on previous studies and new models that accurately reflect the human immune system may improve translational value of research aimed at restoring CD4 T cell-mediated immunity, and overall immune fitness following sepsis.

## Introduction

Sepsis is life-threatening organ dysfunction that results from an exaggerated host immune response to disseminated infection (1). It is characterized (in part) by increased production of both pro- and anti-inflammatory cytokines, resulting in transient severe lymphopenia and long-lasting immune dysfunction (2). Each year at least 1.7 million adult Americans develop sepsis and nearly 270,000 Americans die as a result of sepsis (3). Hospital costs associated with treating sepsis total >$23 billion each year, making it the most expensive condition treated in the U.S. (4). Due to advances in medical care, the majority (~75%) of today's septic patients survive the cytokine storm that results from the initial septic event (5). However, surviving patients suffer from a long-lasting state of immune dysfunction termed immunoparalysis and display increased susceptibility to secondary infection, increased viral reactivation, and decreased 5-year survival compared to individuals who did not develop sepsis (6–8).

The first signs of immunoparalysis can be seen during and shortly after resolution of the cytokine storm in the numerical loss of many cell types, but most notably lymphocytes (9). Lymphocyte numbers recover after resolution of the cytokine storm, but the functional capacity of lymphocytes that reconstitute the immune system is impaired for an extended period (10). Therefore, experimental therapies aimed at alleviating sepsis-induced immunoparalysis have focused on reducing cell loss, increasing numerical recovery, and restoring function of cells that repopulate the immune system (11). Experimental mouse models have been instrumental in informing our knowledge of the impact of sepsis on the immune system and the benefits of perspective therapies for promoting recovery of immune cell numbers and function. However, the translational value of mouse studies depends on how accurately they reflect the human condition (12, 13), and recent studies have highlighted how some aspects of the immune response in inbred, SPF mice do not accurately reflect the immune response in the outbred, non-SPF human population. For example, studies conducted in outbred Swiss Webster mice have shown how inbred mice fail to reflect variation in immune outcomes seen in a genetically diverse population more similar to the human population (14–16). Additionally, studies using microbially-experienced pet store mice or laboratory mice cohoused with pet store mice (a.k.a. “dirty mice”) have shown that exposure to a diverse array of pathogens shapes the immune system. Notably, in contrast to SPF mice that possess an immune system more similar to infants, the immune system of dirty mice more closely resembles that of adult humans (17–21). These studies suggest that incorporating genetic diversity and/or a history of diverse pathogen exposures may improve the translational value of experimental models.

This review will focus on our understanding of how CD4 T cells are impacted by sepsis and how changes within the CD4 T cell compartment affect overall immune fitness. To provide context for this, we will begin with an overview of the effects of sepsis on immune cell subsets, and end with a discussion of therapeutic strategies to alleviate sepsis-induced immunoparalysis, and implications of recent mouse studies that more accurately model sepsis in humans.

## Effects of Sepsis On Immune Cell Subsets

**Figure 1:**
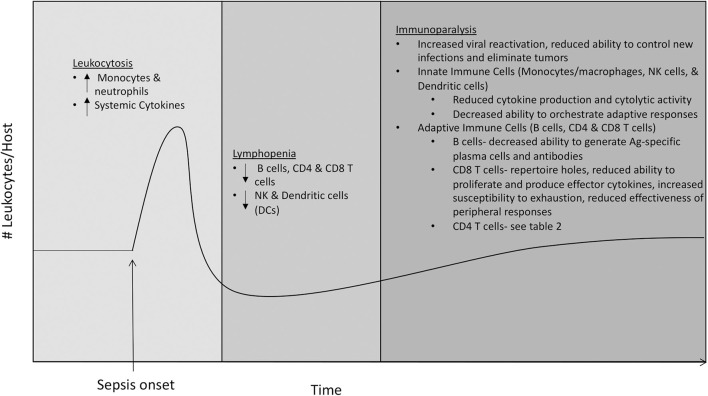
Effects of sepsis on immune cell subsets. The immune system enters a state of leukocytosis during the first 2−4 days following sepsis onset, with marked increases in neutrophil and monocyte populations and increased levels of circulating pro- and anti-inflammatory cytokines. The state of leukocytosis is followed by a state of lymphopenia, characterized by a marked decrease in numbers of adaptive immune cells including B cells, CD4 and CD8 T cells, and innate immune cells including NK cells and dendritic cells (DCs). The state of lymphopenia resolves ~1 month after sepsis onset, as numbers of leukocytes return to normal. Despite the numerical recovery of immune cells, hosts that have recovered from sepsis suffer from a long-lasting state of immune dysfunction termed immunoparalysis. The state of immunoparalysis is characterized by reduced functionality of both innate and adaptive immune cells, increased viral reactivation, and reduced ability to control new infections and to eliminate solid tumors.

Sepsis causes a seismic shift in representation and function of immune cell subsets (Figure 1), which contributes to both the pathophysiology of sepsis and resulting immunoparalysis. Sepsis is initially characterized by leukocytosis in the first 2–4 days, with marked increases in neutrophil and monocyte populations, which is followed quickly by a state of lymphopenia (22, 23). Lymphocyte populations are especially susceptible to apoptosis, and numbers of B cells and CD4 and CD8 T cells are markedly reduced following sepsis onset (9, 23–29). Failure to normalize cell numbers during either the stages of leukocytosis or lymphopenia is associated with increased mortality. In surviving patients, cell numbers return to normal within a month, but failure to prevent viral reactivation and reduced effectiveness at handling new infections suggests long-lasting functional impairments (6–8).

Due to the important roles they play in initial pathogen recognition and response and orchestration of adaptive immune responses, defects in innate immune cells including monocytes/macrophages, neutrophils, NK cells, and dendritic cells (DCs) greatly impact overall immune fitness. Unlike monocytes/macrophages and neutrophils, numbers and on-per-cell basis function of NK cells and DCs initially decline following sepsis (9, 23, 30, 31). RNA-sequencing has revealed that multiple immune-response pathways are down-regulated in monocytes of sepsis patients (32), and mass cytometry (CyTOF), which allows for simultaneous analysis of more parameters than conventional flow cytometry, has shown that monocytes of sepsis patients have increased expression of the inhibitory ligand PD-L1 and decreased expression of HLA-DR (33). Considering that increased expression of inhibitory molecules BTLA and PD-1 on monocytes/macrophages following sepsis has been shown to impact bacterial clearance (34, 35), these findings suggest that alterations in monocytes/macrophages contribute to defective host innate immunity resulting from sepsis. Additionally, decreased expression of HLA-DR could reduce the ability of monocytes/macrophages to present antigen (Ag) and prime B and T cell responses, so these data also suggest that alterations in monocytes/macrophages may also contribute defective host adaptive immunity resulting from sepsis. NK cells that remain following sepsis have a reduced ability to produce the effector cytokine IFN-γ in response to inflammatory cytokines IL-12 and IL-18 or following infection, as well as the reduced ability to degranulate and execute cytolytic activity following Ly49H receptor-mediated activation. Consequently, these numerical and functional defects resulting from sepsis lead to decreased NK cell-mediated control of viral infection (30). In addition, DCs present following sepsis have a decreased ability to produce “signal 3” cytokines (e.g., IFN-γ) in response to TLR stimulation or pathogen challenge, and to prime T cell responses (31, 36). Taken together, these studies suggest that defects in innate immune cell subsets following sepsis contribute to immunoparalysis through both reduced innate antimicrobial activity and decreased ability to stimulate adaptive immune responses (Figure 1).

In addition to quantitative and qualitative alterations in multiple innate immune cell populations, it has become clear that cell-intrinsic defects in B cells and T cells also persist following sepsis (Figure 1). Sepsis results in reduced representation of immature B cells and increased representation of mature B cells, with increased numbers of plasma cells and shifts in representation of B1 and B2 B cells (29, 36, 37). Despite increased plasma cell numbers, Ag-specific antibody production is impaired following sepsis (36–38), suggesting sepsis decreases host ability to develop Ag-specific plasma cells. Following sepsis, CD8 T cells have a reduced ability to prevent infection (39), which is likely due to many factors. Recovery of naïve CD8 T cells following sepsis is incomplete, resulting in loss of some precursor specificities and inability to form responses to some newly encountered Ags (40). The memory CD8 T cells that remain following sepsis display defects in Ag-dependent and -independent functions including reduced Ag-sensitivity, proliferative capacity, and ability to produce cytokines in a bystander manner (41). Furthermore, memory CD8 T cells from hosts that have recovered from sepsis are more prone to undergo exhaustion when combating chronic infections, displaying increased expression of inhibitory receptors PD-1 and 2B4, reduced ability to produce effector cytokines IFN-γ and TNF-α, and reduced ability to clear the infection (42–44). Interestingly, numerical loss and functional defects are not as profound for infection-induced tissue resident memory (T_RM_) CD8 T cells in hosts that survive sepsis. However, immune responses initiated by CD8 T_RM_ from septic hosts are still ineffective due to the inability of endothelial cells to transmit alarm signals, resulting in reduced recruitment of circulating effector cells to the site of infection (45). Decreased protective capacity of CD8 T cells following sepsis extends beyond pathogenic infection, as tumor-infiltrating CD8 T cells from septic hosts have reduced ability to proliferate, produce IFN-γ, and prevent tumor growth (46). However, CD8 T cells from tumor-bearing hosts that experienced sepsis, under certain conditions, could be even reinvigorated due to sepsis-induced release of tumor Ags, leading to the surprising reduction in tumor burden (47). Many defects in CD4 T cells have also been found, and due to their role in providing help to B cells and CD8 T cells, we will discuss the effects of sepsis on CD4 T cells in further detail in the following section.

## Effects of Sepsis On CD4 T Cells

### CD4 T Cell Loss, Recovery, and Repertoire Changes Following Sepsis

Numbers of CD4 T cells are greatly reduced following sepsis onset (24–27, 48–50). Absolute CD4 T cell numbers return to pre-septic levels after a month for most patients, but failure to recover sufficient numbers of immunocompetent CD4 T cells is associated with poor prognosis, especially in the elderly (24, 27, 49, 50). However, questions remain as to how numerical recovery of CD4 T cells occurs and the roles that thymic output, homeostatic proliferation, and Ag-driven proliferation play in that recovery. Initial experiments examining numerical recovery of CD4 T cells showed increased percentages of CD4 T cells expressing markers associated with memory (e.g., CD44^hi^, CD62L^low^) following sepsis, suggesting recovery occurs through homeostatic proliferation of naïve cells, Ag-driven proliferation, and/or outgrowth of endogenous memory CD4 T cell populations (49). However, the authors found that adoptively transferred naïve TCR-transgenic OT-II CD4 T cells did not proliferate when transferred into septic hosts suggesting CD4 T cell recovery does not occur through homeostatic proliferation. Additionally, no skewing in TCR Vβ expression in memory CD4 T cells following sepsis was observed, suggesting numerical recovery was not due to Ag-driven proliferation of cells responding to infection during sepsis. By ruling out homeostatic proliferation and Ag-driven proliferation, the authors concluded that numerical recovery results from outgrowth of endogenous memory CD4 T cells—even though this conclusion was not formally proven in this study. In contrast, a later study found decreased TCR Vβ diversity in human sepsis patients, which was associated with increased risk of death (51). Data published from our group (50) found CD4 T cell numerical recovery occurred similarly in wild type and thymectomized mice, suggesting numerical recovery occurs independently of thymic output. As in previous studies, numerical recovery of CD4 T cells was accompanied by accumulation of cells with an Ag-experienced phenotype (i.e., upregulation of CD11a and CD49d). However, both adoptively transferred TCR-transgenic CD4 T cells and endogenous CD4 T cells of known epitope specificity that were present during the septic event (rather than transferred post-sepsis) proliferated in septic hosts, suggesting that numerical recovery of CD4 T cells is driven at least in part by homeostatic proliferation. Ag-driven proliferation also is likely to play a role for some Ag-specific CD4 T cell populations, as CD4 T cells recognizing epitopes derived from gut-derived segmented filamentous bacterium (SFB) were found to proliferate in an Ag-dependent manner following sepsis (52). Importantly, recovery of epitope-specific CD4 T cells occurred asymmetrically following homeostatic proliferation. When numerical representation of six different Ag-specific CD4 T cell populations was determined in sham and 1 month post-sepsis mice, half of Ag-specific populations recovered numerically, while one population was found in greater numbers and two were numerically reduced post-sepsis (50). Furthermore, Ag-specific populations that failed to recover numerically displayed functional defects including decreased ability to proliferate and to produce cytokines following infection or incubation with cognate Ag and to mount Th17 polarized responses. Thus, changes within the CD4 T cell compartment during numerical recovery (Table 1) impact their ability to respond to newly encountered Ags, which likely impacts their ability to provide protection against newly encountered infections.

**Table 1:** Effects of sepsis on CD4 T cells.

**Category**	**Effects**	**References**
Repertoire changes	Decreased TCR Vβ diversity in humans	(51)
	Incomplete recovery of some epitope specificities	(50)
	Ag-dependent proliferation for some specificities	(52)
Functional defects	Impaired DTH responses and higher rates of viral reactivation	(53–55)
	Global anergy	
	•Reduced ability to produce cytokines •Reduced ability to proliferate •Increased expression of inhibitory receptors	(2, 56–61) (50, 56, 62) (34, 35, 63–68)
Changes in subset representation	Decreased transcript levels of T-bet, GATA3, and ROR-γT	(69)
	Repressive histone methylation at IFN-γ and GATA3 promoter regions	(62)
	Increased Treg cell representation	(26, 59, 70, 71)
	Decreased representation of Th1, Th2, Th17, and Tfh subsets	(28, 59, 71, 72)

### CD4 T Cell Functional Defects Following Sepsis

Evidence for functional defects of CD4 T cells in septic patients was first inferred from studies showing impaired DTH skin reactions (53). Later studies pointed to the significantly higher rates of CMV and HSV reactivation in septic patients (54, 55)—infections for which effective CD4 T cell immunity is essential for limiting frequency and severity of recrudescence in humans (54, 73–75). Early studies that examined cytokine production by CD4 T cells from septic patients showed that cytokines produced under Th1 or Th2 conditions were altered (56–60), leading to the suggestion that sepsis caused a phenotypic switch of CD4 T cells from Th1 to Th2 (61). However, a later study examining cytokine production by freshly isolated, postmortem spleen and lung samples found almost no production of IFN-γ, TNF-α, IL-6, and IL-10 after anti-CD3/CD28 mAb stimulation (2), providing evidence for the suggestion that post-septic CD4 T cells display a global state of anergy (56). This argument was strengthened by studies showing reduced proliferative capacity; decreased mRNA transcript levels of T-bet, GATA3, and ROR-γt transcription factors that regulate differentiation into Th1, Th2, and Th17 CD4 T cell subsets, respectively; and repressive histone methylation marks at the IFN-γ and GATA-3 promoter regions of CD4 T cells taken from septic hosts (50, 62, 69). Decreased ability to proliferate and produce effector cytokines is reminiscent of functional defects arising during T cell exhaustion caused by prolonged antigen exposure and inflammation in the face of chronic viral infection and cancer (76–78). Exhaustion is accompanied by increased expression of inhibitory receptors that dampen immune responses, and CD4 T cells from septic hosts have greater expression of inhibitory receptors including PD-1, 2B4, BTLA, and TRAIL, which directly impacts their ability to effectively respond to infection (34, 35, 63–68). Furthermore, expression of inhibitory receptors has the potential to impact CD4 T cell-derived help to other cells, including B cells and T cells. In support of this, reduced effectiveness of CD8 T cell immune responses in septic hosts has been shown to be due in part to TRAIL-dependent mechanisms (67, 68, 79). Thus, sepsis causes global changes in expression of factors regulating CD4 T cell effector responses (Table 1), which limits help provided to other immune cells and effectiveness of *de novo* immune responses.

It should be noted, however, that triggering events and microorganisms capable of inducing sepsis are numerous. The most common triggering event in humans is pulmonary infection, with other common triggers including infections of the abdomen (e.g., those arising from a perforated or ischemic bowel), soft tissues (often as a result of burns), and the urinary tract (80, 81). Microorganisms that commonly cause sepsis include gram-positive (*Staphylococcus aureus* and *Streptococcus pneumoniae*) and gram-negative (*Escherichia coli* and *Klebsiella* species) bacteria, fungal organisms, and viruses including SARS-CoV-2 (82–85). Triggering events and causative microbes for studies that suggested CD4 T cells from recovered sepsis patients exist in a state of global anergy varied among patients (2). It is unclear if or how different triggering events or factors unique to the causative pathogens, such as their mitogenic capacity or quality and/or severity of the cytokine storm they elicit, influence the severity of CD4 T cell functional defects observed in patients who have recovered from sepsis.

### Changes in CD4 T Cell Subsets Following Sepsis

One of the defining features of CD4 T cells is that they are able to differentiate into subsets capable of performing unique effector functions best suited to drive responses against perceived threats based upon polarizing inflammatory cytokine and co-stimulatory molecule signals present during Ag-presentation. Based on the literature, it is clear that sepsis disrupts both representation of and function of CD4 T cell subsets, including Th1, Th2, Th17, Tfh, and Treg subsets (Table 1). A number of studies have noted an increased frequency of Treg cells in the periphery of septic patients (26, 70, 71), which was later shown to be the result of preferential loss of other subsets (i.e., Th1, Th2, Th17, and Tfh) (28, 59, 71, 72, 86). It should be noted, however, that these observations in humans are based upon analysis of cells found in the blood. Considering that mouse studies have shown lymphocytes in tissues are less susceptible to sepsis-induced alterations (28, 45), similar shifts in CD4 T cell subset representation may not be observed in peripheral tissues of humans. Losses in CD4 T cell subsets impacts CD4 T cell-mediated help provided to other cell types, as was recently demonstrated for reduced antibody production resulting from CD4 T cell-dependent B cell responses, which was caused in part by reduced Tfh differentiation following immunization of septic hosts (38). In addition, the effects of sepsis on the ability to produce effector cytokines (IL-10 in the case of Treg) may be less severe for Treg than for other CD4 T cell subsets (87). The impact of increased Treg cell representation following sepsis has been debated, as some have correlated it with worse outcomes (88), while others have suggested it correlates with better outcomes and immunity (89–91). Studies using anti-GITR mAb to block Treg function (92) and siRNA to downregulate Foxp3 expression (93) showed that reducing Treg numbers and/or function in septic hosts improved overall immune function and pathogen control. However, later studies concluded that depletion of Tregs did not lead to improvements in survival (94), although interpretation of this study is compounded by the use of anti-CD25 mAb, which can deplete CD25-expressing cells (such as effector T cells) other than Tregs. In addition to the factors mentioned above, discrepancies for the role of Treg cells in sepsis pathology and immunoparalysis may be due to timing of analysis, as a recent study has suggested Treg cells contribute to positive outcomes during the early stages of sepsis, but do not significantly impact immunosuppression seen following recovery (95). Regardless, the continued debate concerning the role Treg cells play in sepsis pathology and immunoparalysis calls for a more detailed analysis.

If targeting changes in CD4 T cell subset representation could provide a therapeutic benefit to sepsis patients, understanding the factors leading to these imbalances becomes important. Altered functions and loss of other immune cell subsets likely plays a role in the remodeling of CD4 T cell subsets following sepsis. Adoptive transfer of bone marrow-derived DCs (BMDCs) to septic animals elevated levels of Th1 cytokines, reduced expression of the inhibitory receptor PD-1 on CD4 T cells, reduced proliferation and differentiation of Treg cells, and increased rates of survival (96). Additionally, IL-33—a cytokine that plays a role in promoting Treg expansion—is elevated in septic patients, and recent studies showed neutralization of IL-33 limited the immunosuppressive effects of sepsis and improved outcomes following secondary infection (97). These studies suggest therapies designed to restore numbers and function of immune cells other than CD4 T cells may be beneficial for reestablishing the balance of CD4 T cell subsets following sepsis and for reducing the effects of increased Treg representation. Furthermore, it is becoming appreciated that sepsis alters the metabolic capacity of T cells (98), and targeting the effects of sepsis on immunometabolism presents an intriguing opportunity to restore T cell dysfunction resulting from sepsis. Targeting metabolism may help to prevent undesirable shifts in CD4 T cell subsets following sepsis, based on recent data showing administration of glutamine led to decreased representation of Th2 and Treg cells in septic hosts (99). While there is much work to be done to fully understand how changes in CD4 T cell subsets observed following sepsis impact the state of immunoparalysis, these studies present the exciting possibility that therapies may be developed to limit CD4 T cell subset alterations following sepsis and promote restoration of protective T cell immunity.

## Experimental Therapies to Alleviate Sepsis-Induced Immunoparalysis

Due to the contributions of numerical cell loss and functional defects, therapies designed to alleviate sepsis-induced immunoparalysis have focused on reducing cell death, expanding numbers of surviving cells, and restoring function of those cells. Initial experiments designed to block apoptosis through overexpression of the antiapoptotic molecule Bcl-2 or inhibition of caspases showed a clear survival benefit for septic hosts (100–103). However, the use of caspase inhibitors to treat sepsis was not widely adopted due to the importance of caspases to other cellular processes and difficulties in establishing doses and timing of administration that provided clinical benefit. Because of this, the most promising strategies currently involve single or combination therapies with γc receptor-dependent cytokines and blockade of inhibitory molecules, which both have the potential to increase cell numbers and restore cell functions.

Common γc cytokines, including IL-2, IL-7, and IL-15, promote the survival of naïve, effector, and memory CD4 and CD8 T cells. While IL-2 and IL-15 have shown therapeutic benefits (104–106), indicating that further exploration of their use in treatment of sepsis is warranted, therapeutic administration of IL-7 is well-tolerated and shows promise to reverse immunoparalysis of sepsis patients. Studies conducted over the last several years have shown that IL-7 administration improves T cell survival; functionality of surviving T cells including ability to proliferate, traffic, and to produce effector cytokines including IFN-γ, TNF-α, and IL-17; and ability to stimulate DTH responses and clear secondary infections (107–110). IL-7 treatment may also help to restore metabolic defects of T cells present after sepsis recovery, as IL-7 was recently shown to promote activation of mTOR, an important regulator of oxidative phosphorylation, in T cells of sepsis patients (108). Importantly, recent results from clinical trials have shown IL-7 administration is well-tolerated in sepsis patients and results in improved numbers and functions of CD4 and CD8 T cells (110), pointing to the translational value of this treatment for sepsis patients. It will be important to follow septic patients treated with IL-7 in the future to see if improvements in immune cell numbers and functions translate to improved ability to prevent opportunistic secondary infections and better long-term outcomes.

Interactions between inhibitory receptors, such as PD-1, CTLA-4, BTLA, Tim-3, LAG-3, 2B4, and TRAIL expressed by T cells and their cognate ligands can be generally described to have inhibitory effects on T cell function. Immune checkpoint modulation therapy, which is used to block interactions of inhibitory receptors and their ligands, has shown great promise for reducing functional defects of exhausted T cells in settings of chronic infection and as a therapeutic treatment of certain cancers (111–113). Because T cells of sepsis patients share such similarities to exhausted T cells, including increased expression of inhibitory receptors and functional anergy (34, 35, 63–68), immune checkpoint modulation has been explored as a therapeutic strategy to reverse sepsis-induced immunosuppression. Therapeutic administration of agents blocking inhibitory receptor interactions of PD-1/PDL-1, 2B4, Tim-3, CTLA-4, LAG-3, and TRAIL have all shown some benefit for improving function of T cells and monocytes of septic hosts, including improving expression of the costimulatory molecule CD28, ability of T cells and macrophages to produce inflammatory cytokines, and ability of CD8 T cells to form memory populations (68, 107, 114–123). However, the immunomodulatory effects of treatments targeting immune checkpoint pathways in septic hosts are dependent upon dose and timing of administration (116, 117), which will require careful consideration for clinical use. Additionally, treatments based on administration of IL-7 and PD-1 blockade have differing effects on reversing sepsis-induced immunosuppression (107), suggesting that combined treatments may have synergistic effects. While their long-term effects on restoring fully protective immune responses of septic patients remain to be elucidated, improvements in immune cell numbers and function following administration of γc cytokines and checkpoint blockade inhibitors are promising signs for their use as therapies to reverse immunoparalysis resulting from sepsis.

## Advancement of Animal Models That More Accurately Reflect Sepsis in Humans

While mouse-based preclinical studies have resulted in development of therapies that have shown great efficacy in the clinic, such as the immune checkpoint blockade therapies for the treatment of some cancers, it has also been argued that differences between mice and humans are a major reason for the inability to translate therapies described in laboratories to successful clinical outcomes (124–128). Therefore, developing experimental mouse models that more closely resemble the human condition may improve the translational potential of preclinical sepsis studies. One of the major differences between mouse studies and humans is that the majority of preclinical mouse studies are conducted using inbred mice, which does not reflect the genetic diversity present in the human population. We know from human sepsis studies that outcomes, including survival and resulting parameters of immunoparalysis, vary greatly from person to person (129). While this may be due to a number of factors including patient age, severity of sepsis, and underlying health conditions, genetics may also play a role. Studies utilizing outbred mice have shown inbred mice fail to capture diversity of immune outcomes seen in genetically diverse populations (14–16). Only a limited number of sepsis studies have included outbred mice and/or mice of varied genetic background, but these experiments have provided insight into how models of sepsis in mice might compare to outcomes in humans. Studies using outbred Swiss mice have shown that immunoparalysis following sepsis, including reduced numbers and function of both DCs and CD8 T cells, can be observed in outbred as well as inbred mice (31, 41, 42, 45), suggesting some aspects of immunoparalysis are likely to be universal in a population of mixed genetics. However, other parameters of immunoparalysis might differ based in part on genetics, as the percentage of MHC II-expressing lymphocytes and representation of Treg cells post-sepsis was found to differ between BALB/c and outbred CD-1 mice (130). Thus, use of genetically diverse mice in sepsis studies should be encouraged, as they could help uncover aspects of sepsis that are influenced by genetics, as well as help to pinpoint genetic factors responsible for divergent sepsis outcomes in the human population.

Another big difference between mouse studies, which are primarily conducted using SPF mice, and humans is that humans are exposed to a diverse array of pathogens throughout life. Recent studies have shown that the immune system of SPF mice is more similar to human infants, while “dirty” mice that have been exposed to a diverse array of pathogens through co-housing with pet store mice possess an immune system more similar to adult humans (17–21). Importantly, the training and shaping of the immune system that occurred as a result of pathogen exposure rendered mice less susceptible to newly encountered infections, suggesting the history of infection may also influence how organisms respond to a septic insult. Recent work from our laboratory, however, found that microbial exposure results in an enhanced cytokine storm following sepsis and increases risk of mortality (131). While changes in the microbiome due to cohousing were partially responsible for this outcome, changes in function of immune cells due to history of pathogen encounter also played a role, as leukocytes of cohoused mice displayed increased expression of TLR4 and produced greater amounts of inflammatory cytokines in response to LPS. Thus, changes in the immune system due to history of infection with diverse pathogens, which varies from person to person shape the response to septic insult. This also could impact the effectiveness of treatments for sepsis, as antibiotic treatment of septic hosts possessing pre-established memory populations was more effective when combined with memory cell reactivation (132). Mouse models that incorporate a history of pathogen exposure also may improve translatability of sepsis studies, as was recently demonstrated using laboratory mice born to wild mice, which possess similar microbiota and history of pathogen exposure to dams (21). Using this model, the authors were able to replicate clinical trial data showing TNF-α neutralization was ineffective in their dirty mice (just like in human sepsis patients), even though it was an effective therapy for SPF mice. Clearly, increased use of mouse models that incorporate history of pathogen exposure have the potential to increase our understanding of sepsis pathology and resulting immunoparalysis in humans, and to improve translatability of sepsis studies that utilize animals.

## Conclusions

Advancing therapies to reverse sepsis-induced immunoparalysis will require a thorough understanding of defects in immune cell subsets resulting from sepsis, and how those defects contribute to decreased host immune fitness. CD4 T cells play an important role in orchestrating successful immune responses due to their ability to provide help to a range of immune cell types. Therefore, understanding how CD4 T cells are impacted by sepsis, including numerical and functional alterations and changes in subset representation, is an important goal in sepsis-based research. Mouse models that more closely represent the human condition through incorporation of host genetic differences and history of infection with diverse pathogens have the potential to increase our understanding of defects in immune cells of various types caused by sepsis and to improve the translational value of animal-based sepsis studies.

## Author Contributions

All authors listed have made a substantial, direct and intellectual contribution to the work, and approved it for publication.

## Conflict of Interest

The authors declare that the research was conducted in the absence of any commercial or financial relationships that could be construed as a potential conflict of interest.
